# Influence of Conditioning Temperature on Defects in the Double Al_2_O_3_/ZnO Layer Deposited by the ALD Method

**DOI:** 10.3390/ma14041038

**Published:** 2021-02-22

**Authors:** Katarzyna Gawlińska-Nęcek, Mateusz Wlazło, Robert Socha, Ireneusz Stefaniuk, Łukasz Major, Piotr Panek

**Affiliations:** 1 Institute of Metallurgy and Materials Science PAS, Reymonta 25, 30-059 Krakow, Poland; l.major@imim.pl (Ł.M.); p.panek@imim.pl (P.P.); 2 CBRTP—Research and Development Center of Technology for Industry, Ludwika Waryńskiego 3A, 00-645 Warszawa, Poland; mateusz.wlazlo@cbrtp.pl (M.W.); ncsocha@cyf-kr.edu.pl (R.S.); 3 Institute of Catalysis and Surface Chemistry PAS, Niezapominajek 8, 30-239 Krakow, Poland; 4 Center of Teaching Technical and Natural Sciences, University of Rzeszow, Pigonia 1, 35-959 Rzeszow, Poland; istef@ur.edu.pl

**Keywords:** semiconductor-insulator interfaces, interface phenomena, Al_2_O_3_/ZnO double layer, heterojunction solar cell, ALD, defects analysis, EPR

## Abstract

In this work, we present the results of defects analysis concerning ZnO and Al_2_O_3_ layers deposited by atomic layer deposition (ALD) technique. The analysis was performed by the X-band electron paramagnetic resonance (EPR) spectroscopy, transmission electron microscopy (TEM) and X-ray photoelectron spectroscopy (XPS) methods. The layers were either tested as-deposited or after 30 min heating at 300 °C and 450 °C in Ar atmosphere. TEM and XPS investigations revealed amorphous nature and non-stoichiometry of aluminum oxide even after additional high-temperature treatment. EPR confirmed high number of defect states in Al_2_O_3_. For ZnO, we found the as-deposited layer shows ultrafine grains that start to grow when high temperature is applied and that their crystallinity is also improved, resulting in good agreement with XPS results which indicated lower number of defects on the layer surface.

## 1. Introduction

An important research area in the field of photovoltaics is the use of cheap, abundant and non-toxic materials for the production of solar cells. Metal oxides with a wide range of electronic properties find application in photovoltaic technology, including semiconducting oxides (such as ZnO, TiO_2_), insulators (such as Al_2_O_3_), or transparent conductive oxides, such as ZnO:Al (AZO) or In_2_O_3_:Sn (ITO). The possibility of practical application of metal oxides in photovoltaics is determined by their basic parameters such as reflection coefficient (R), transmission coefficient (T), resistivity (ρ), band gap energy (Eg), location of valence and conduction bands, charge carrier mobility (µ), work function (ϕ) and conductivity type. Depending on the exact values of these parameters, metal oxides can act as passivating layers, anti-reflective coatings, tunneling layers, insulators, or even replace a metal contact [1]. Additionally, metal oxides with different types of conductivity can form a heterojunction with both p- and n-type silicon [2] or with other oxides [3,4].

One of the most widely used conductive oxides in electronics is zinc oxide (ZnO) [5], an n-type semiconductor with a band gap energy 3.37 eV and an exciton binding energy at room temperature of 60 meV [6]. ZnO crystallizes in two main forms, hexagonal wurtzite (B4) and cubic zincblende. The first one is more stable in ambient conditions [7]. In hexagonal ZnO, the crystal structure is described by the space group P63mc. It is characterized by two interconnecting tetrahedrally-coordinated Zn^2+^ and O^2−^ lattices. This configuration gives a polar symmetry along the sixfold axis and enhances oxygen vacancies (V_O_) and zinc interstitials (i_Zn_) defect generation. The typical resistivity of ZnO is 1–100 Ωcm but it can be reduced to as low as 10^−4^ Ωcm with electron mobility of 200 cm^2^/Vs when a dopant is introduced [8]. Due to a high value of light transmission coefficient and the ability to control resistivity, zinc oxide becomes a competitive material for expensive ITO layers used in optoelectronics [1,5,9]. Moreover, it also finds application in other products, such as hydrogen [10] or pressure sensors [11].

Another interesting oxide is Al_2_O_3_, widely used for its insulating properties. It often acts as a passivation layer in solar cells [12,13]. Aluminum oxide deposited on a top of p-type silicon by atomic layer deposition (ALD) method and heated at 370 °C in H_2_/N_2_ atmosphere reduces its surface recombination velocity to 10 cm/s [14].

Both ZnO and Al_2_O_3_ can be manufactured by many methods such as spin coating [15], molecular beam epitaxy [16], sol-gel [17], electrodeposition [18], microwave-assisted growth [19], sonochemical method [20] and hydrothermal method [21]. Another technique of thin layer manufacturing is ALD [22], which has a few advantages over other physical and chemical deposition techniques. Among them are precise composition and thickness control, high level of conformity and uniformity, even on non-planar substrates, and self-limiting character of the chemical process between the substrate and precursors. The study of surface phenomena at the ZnO-Al_2_O_3_ semiconductor-insulator interface is crucial for understanding interface effects occurring in such a junction. In nanostructured systems where the layers are deposited in a patterned manner, such as in the form of nanowires/nanorods [23], the interface has a high surface area and thus, the surface effects play a bigger role than in the case of the flat interface. For a good interface, surface morphology with a low film roughness is required. ALD is a technique that ensures conformal and uniform growth [24]. The resulting surface is, in principle, atomically flat. In practice, having conformal growth means that any surface features from the underlying layer will be reproduced. This is especially true for nanometer-scale films.

In the present study, we investigated the Al_2_O_3_/ZnO layers’ arrangement with the target application in heterojunction structure of p-Si/Al_2_O_3_/n-ZnO where Al_2_O_3_ is used as passivation layer for interface defects with tunneling function in the case of working solar cell. Both layers were deposited by the ALD method and they were fired at different temperatures in protective argon (Ar) atmosphere. The presence of defects was analyzed by X-band electron paramagnetic resonance (EPR) spectroscopy, transmission electron microscopy (TEM) and X-ray photoelectron spectroscopy (XPS) methods. The novelty aspect of the work is the study of the influence of temperature treatment on defects in the Al_2_O_3_/ZnO double layer through the correlation of the XPS and EPR results.

## 2. Materials and Methods

**Figure 1:**
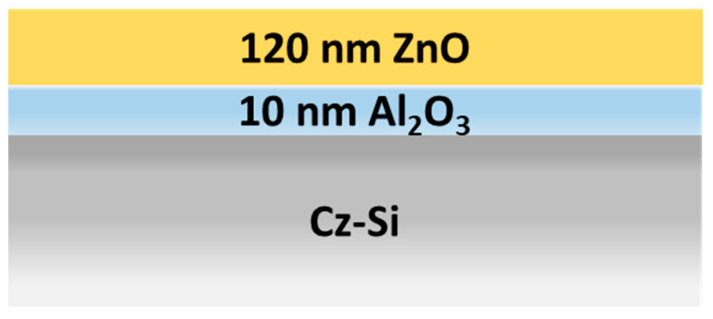
Scheme of the investigated structure.

ZnO and Al_2_O_3_ layers were produced by the atomic layer deposition (ALD) method on a Beneq P400A industrial scale reactor. The Zn and Al precursors were diethyl zinc Zn(C_2_H_5_)_2_ (DEZ, CAS 557-20-0, Lanxess, Cologne, Germany) and trimethylaluminum Al(CH_3_)_3_ (TMA, CAS 75-24-1, Lanxess, Cologne, Germany) respectively. The layers were manufactured at 200 °C in an inert gas (N_2_) atmosphere and with deionized water (H_2_O) as the oxygen precursor. The Al_2_O_3_ and ZnO layers with a nominal thicknesses of 10 nm and 120 nm, respectively, were deposited on the polished p-type Cz-Si wafers’ substrate with crystallographic orientation (100) (Sieger Wafer, Aachen, Germany, J13073), quartz glass substrates (Fused Quartz, JGS-2) and capillary tubes (Bruker, Billerica, MA, USA, ER 221TUB/4 CFQ). The silicon substrates were etched in diluted HF before Al_2_O_3_ layer deposition, in order to remove native silicon oxide present on its surface. After deposition, the samples were removed from the ALD reactor chamber, transferred to a furnace and subjected to annealing process at 300 °C and 450 °C in Ar atmosphere for 30 min. In order to meet the criteria of electrical charge tunneling layer, the Al_2_O_3_ should not be thicker than 1.0–1.5 nm [25]. However, in our investigation a 10 nm-thick Al_2_O_3_ was deposited for higher accuracy of XPS and EPR measurements and better detection of the temperature effect on the layer parameters. The scheme of the investigated structure is shown in Figure 1.

X-ray photoelectron spectroscopy (XPS) in ultra-high vacuum (1 × 10^−9^ mbar) was used to determine the surface composition and chemical coordination of species deposited on glass substrates by ALD. The measurements were carried out with a XPS spectrometer produced by Prevac (Rogów, Poland) with hemispherical SES R4000 analyzer Gammadata Scienta (Uppsala, Sweden). The Al Kα (1486.6 eV) line produced by the X-ray source was applied to generate core excitations. Shirley-type background subtraction was applied for spectral analysis prior to fitting procedure. The Voigt line shape, i.e., Gaussian/Lorentzian function, was used. The surface composition was analyzed with XPS from a relatively large surface area of 3 mm^2^. The analytic depth of the method was ca. 10 nm.

Transmission electron microscopy (TEM) investigations were carried out using a Tecnai F20 200 kV (FEG) of the FEI Company (Eindhoven, Netherlands) microscope, equipped with an EDAX energy dispersive X-ray spectroscopy detector (EDS) (Ametek, Mahwah NJ, USA). Thin foils for TEM investigations were prepared by the focused ion beam (FIB) technique, using a Quanta 200 3D Dual Beam (FIB/SEM) system of the FEI Company (Eindhoven, The Netherlands) equipped with an OmniProbe (Oxford Instruments, High Wycombe, UK) micromanipulator.

The X-band electron paramagnetic resonance (EPR) was performed on Bruker (Bruker, Billerica, MA, USA) multifrequency and multiresonance FT-EPR ELEXSYS E580 spectrometer. The test samples were single Al_2_O_3_, single ZnO and double Al_2_O_3_/ZnO layers deposited on quartz glass capillary tubes.

Sheet resistance of ZnO layers on glass was measured by four point probe SPC-90 made by Industrial Institute of Electronics (PIE), Warsaw, Poland.

## 3. Results and Discussion

The resistivity (ρ) of thin ZnO layer deposited on glass substrates was measured by four point probe. The results presented in Table 1 show that ρ of as-deposited zinc oxide with thickness about 120 nm is equal to 6.8 × 10^−3^ Ωcm, which is similar to the literature data [26]. Resistivity increases as post-treatment temperature is increased. The phenomenon was reported by other authors [27,28], but the fullest explanation can be found in the work of Wisz [29]. Columnar growth of the ZnO layer with temperature is accompanied by increase of the layer resistance. As a result of average distance between the crystal columns increasing, the potential barrier at the grain boundaries rises which leads to higher resistivity in the substrate plane. The measurement of sheet resistance using the four point probe was carried out in the x-y plane, while the growth of the ZnO crystalline layer was in the z-plane. Therefore, the degradation of ZnO conductivity is observed. In our investigation, high temperature lattice scattering is a significant contributing factor to the carrier mobility and, as a consequence, layer resistivity.

**Figure 2:**
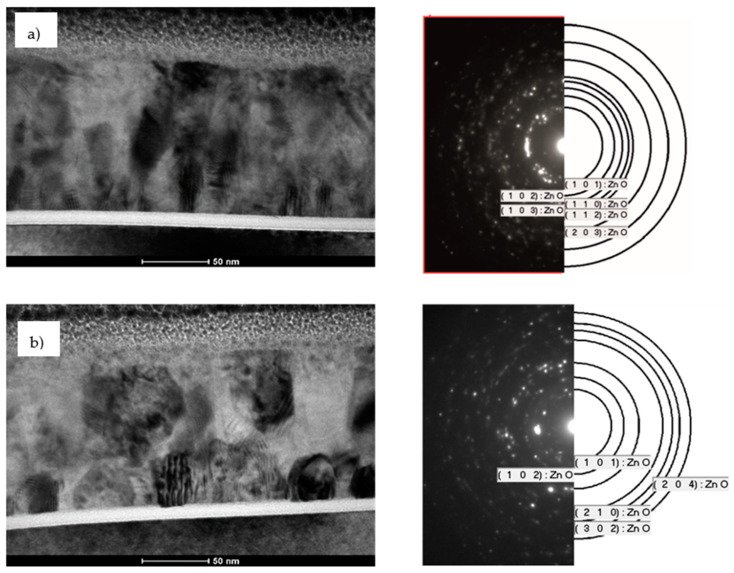
Bright field TEM cross-section together with selected area electron diffraction of (**a**) as-deposited ZnO at 200 °C and (**b**) ZnO after heating at 450 °C for 30 min in Ar atmosphere.

The bright field TEM cross-section analysis revealed that as-deposited ZnO layer (Figure 2a) exhibits grains with an average size of several nanometers. This corresponds to the selected area electron diffraction (SAED) pattern which takes the form of rings created by ultrafine grains of polycrystalline materials with different crystal orientation. Diffraction analysis confirmed presence of only one ZnO phase where the diffraction rings have been fitted to the (101), (102), (103), (110), (112) and (203) crystallographic planes. As shown in Figure 2b, ZnO crystal domains start to grow when heated at 450 °C for 30 min in argon atmosphere. Some of the grains reach even several dozen nanometers. It should be noted that the diameters of heated ZnO domains are smaller near the substrate and much higher in the outer part of the layer. The diffraction pattern confirmed higher crystallinity of heated ZnO which crystallizes, as expected, in a hexagonal structure. Diffraction rings are more fragmented. The fitted crystallographic planes are (101), (102), (204), (210) and (302).

The ZnO layer thickness remains unchanged after annealing, and it is about 120 nm. This suggests that no migration of metal ions occurs upon annealing. The ZnO morphologies remain uniform without any cracks or holes also near the Al_2_O_3_/ZnO interface.

**Figure 3:**
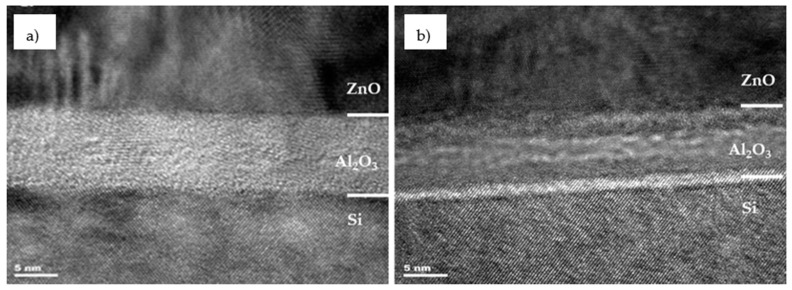
HRTEM cross-section of the Al_2_O_3_/ZnO interface (**a**) after the deposition process at 200 °C, and (**b**) after heating at 450 °C for 30 min in Ar atmosphere.

The morphology of Al_2_O_3_ (Figure 3) was unchanged after heating and it stayed amorphous and continuous. As with ZnO, the thickness of the layer remained unaffected and it is 9 nm. This is lower than the 10 nm thickness determined from ALD recipe. It is worth noticing that for heated Al_2_O_3_, the diffraction contrast appeared and the light and dark area in Al_2_O_3_ volume can be distinguished. The light line is located near to the Si/Al_2_O_3_ interface while the bulk of the Al_2_O_3_ layer is uniformly dark. Presence of high contrast in this area may indicate the diffusion of aluminum into silicon due to the treatment at elevated temperature (450 °C). The slightly lower than expected measured layer thickness may also be a sign of aluminum diffusion into silicon.

**Figure 4:**
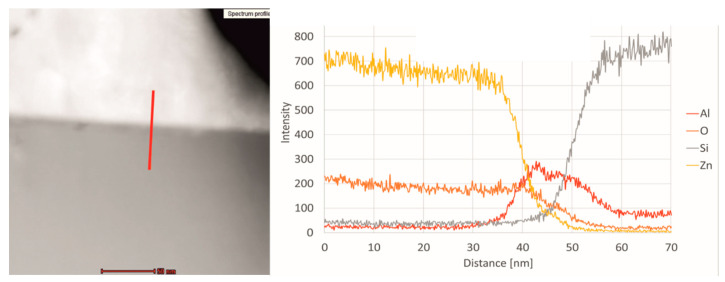
STEM cross-section image of the Si/Al_2_O_3_/ZnO structure heated at 450 °C with EDS line scan analysis from selected area where distance was measured from top to the bottom.

STEM cross-section of Cz-Si/Al_2_O_3_/ZnO structure heated at 450 °C with EDS line scan analysis is presented in Figure 4. The distance of 70 nm was measured from the top of the line to the bottom. The elements’ distribution revealed that the intensity ratio of zinc to oxygen is approximately 7:2 and it starts to decrease when it reaches 36 nm of distance, near to the ZnO/Al_2_O_3_ interface. The aluminum signal appears around 33 nm and its ratio to oxygen is 1:4. Later it starts to increase due to the Al intensity increasing and reaches the 1:1 ratio at 40 nm. Further the O signal falls down while Al signal continues to grow and then stabilizes between 45 and 50 nm. In the same range, the intensity of the silicon line begins to increase, which means that it is the Al_2_O_3_/Si interface region. Significant decrease in the intensity of Al can be observed after reaching 50 nm of analyzed distance. At this depth, the signal coming from the silicon substrate is exhibiting a sharp increase. At 60 nm and above, it dominates the whole spectrum. However, even at a depth of 70 nm, the aluminum signal does not completely vanish as it is the case for oxygen. This result confirms the possibility of diffusion of aluminum into silicon which corresponds to the bright line on HRTEM cross-section of heated ZnO/Al_2_O_3_ (Figure 3b). Such a phenomenon can create a local electric field on the Al_2_O_3_/Si interface, resulting in a p-p^+^ junction [30].

**Figure 5:**
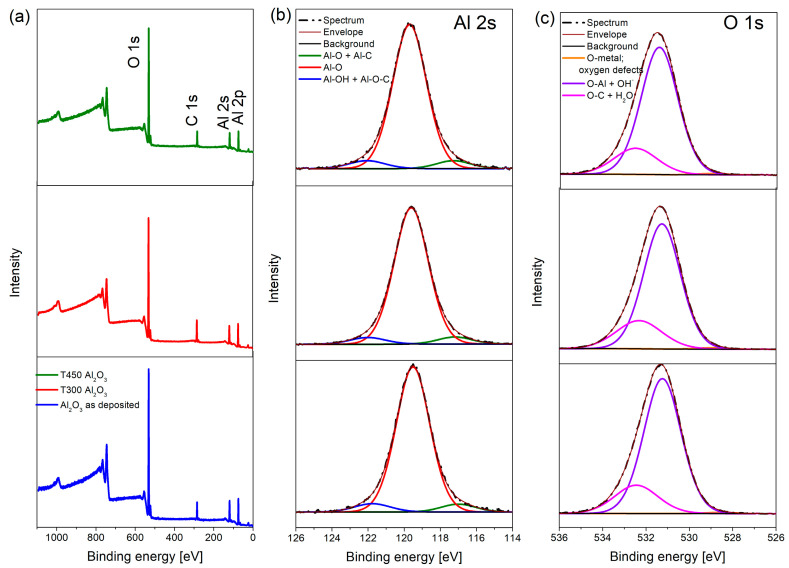
Al_2_O_3_ XPS: (**a**) survey spectra; (**b**) Al 2s spectra; (**c**) O 1s spectra, collected as deposited (**bottom**), after heating at 300 °C (**middle**), and after heating at 450 °C (**top**).

The results of the XPS analysis of single Al_2_O_3_ layer with 10 nm of nominal thickness deposited on glass is presented in Figure 5a. The survey spectra of as-deposited Al_2_O_3_ (blue line), Al_2_O_3_ heated at 300 °C T300 (red line) and Al_2_O_3_ heated at 450 °C T450 (green line) are shown. The calculated Al/O ratio is equal to 0.78 for as-deposited layer, 0.79 for T300 Al_2_O_3_ and 0.74 for T450 Al_2_O_3_. The results suggest off-stoichiometric compositions of all films, otherwise the Al/O ratio would be equal to 0.667. The high-resolution spectra of the two most intensive peaks with binding energy (BE) of 119 eV (Al 2s) and 531 eV (O 1s) are shown in Figure 5b,c. Al 2p spectral analysis (Figure 5b) revealed that the maximum of the most intensive spectrum component, Al 2p_3/2_, is slightly shifted by about 0.1 eV with temperature. It reaches 119.5 eV for as-deposited Al_2_O_3_, 119.6 eV for T300 Al_2_O_3_ and 119.7 eV for T450 Al_2_O_3_. This may indicate a tendency of the layer to transform into α-Al_2_O_3_ with higher crystallinity. Other two components can be ascribed to Al_2_O_4_^2−^, oxide vacancies, lattice defects or Al-C connection (117 eV) and Al-OH or Al-O-C (122 eV). By analyzing the O 1s spectrum (Figure 5c), it can be found that the electronic states of surface oxygen atoms are very similar. The peak at BE 531.2 eV (purple line) can be attributed to oxygen in the metal oxide lattice. Other two components with much lower intensity at BE 532.4 eV (pink line) and 529 eV (orange line) correspond to oxygen in organic carbon compounds or adsorbed water and oxygen vacancies respectively.

**Figure 6:**
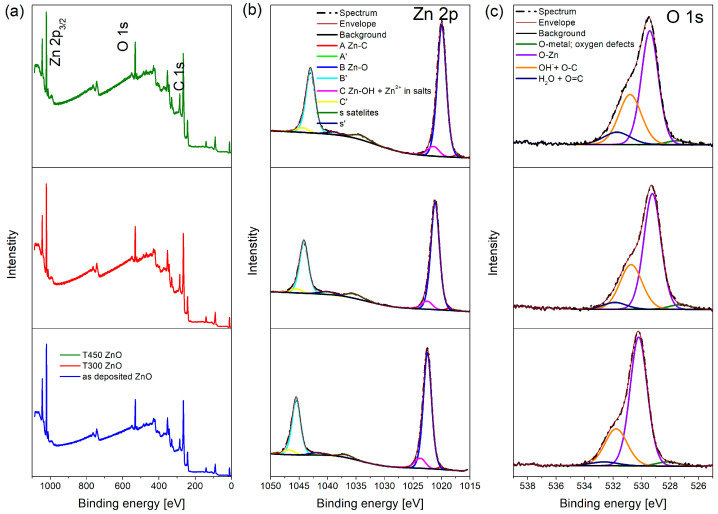
ZnO XPS: (**a**) survey spectra; (**b**) Zn 2p spectra; (**c**) O 1s spectra, collected as deposited (**bottom**), after heating at 300 °C (**middle**), and after heating at 450 °C (**top**).

The survey spectrum of single ZnO layer with a thickness of 120 nm deposited on a glass (Figure 6a) indicates that after annealing, the amount of zinc on the surface decreases. This corresponds to the lower intensity of the peak at BE 1021 eV for T300 ZnO and T450 ZnO. The zinc-to-oxygen atomic ratio decreases from 0.47 for as-deposited ZnO to 0.34 for T300 ZnO and 0.28 for T450 ZnO. It is a result of carbon content increase on the ZnO surface with temperature which is a remnant of an organic precursor. Analysis shows that carbon-to-oxygen ratio grows from 1.64 (as-deposited ZnO) to 1.72 (T300 ZnO) and 1.86 (T450 ZnO). Figure 6b,c present high-resolution spectra of Zn 2p and O 1s peaks respectively. For Zn 2p3/2 core excitation, the most intensive component (blue line) can be assigned to zinc in ZnO lattice. Furthermore, the shift in BE of this peak increases with temperature form 1021 eV for as-deposited ZnO, 1021.1 eV for T300 ZnO and 1021.4 eV for T450 ZnO. This, together with the increasing of Full Width at Half Maximum (FWHM) from 1.70 eV by 1.77 eV to 1.85 eV, respectively, suggests changes in surface crystallinity related to the applied temperature. For highly crystalline ZnO, the BE should be closer to the literature value of 1021.8 eV [31]. Therefore, when both BE and FWHM parameters are considered, we can conclude that the studied surfaces show a number of defects increasing with the temperature of annealing. Other components of the Zn 2p spectrum can be identified as Zn^2+^ ions in salt or hydroxyl surrounding (pink line) and the Zn-C bond (very small red line peak). For O 1s spectrum (Figure 6c), the most intensive peak at BE of 530 eV can be assigned to oxygen in Zn-O. Second component at 531.7 eV is described to oxygen in hydroxyl group or aliphatic compounds (orange line). The BE of 528 eV corresponds to the oxygen vacancies (green line). The final line at 532.7 eV can be identified as oxygen in adsorbed water and longer aliphatic chains. It can be concluded that increasing temperature of the treatment results in increasing number of oxygen vacancies which corroborates the conclusions on Zn 2p line analysis.

Defects analysis in the Al_2_O_3_ and ZnO layers were carried out using the electron paramagnetic resonance method. The EPR method is used to study the location and orientation of paramagnetic point defects in solids. It is based on the phenomenon of resonance absorption of the quantum of electromagnetic radiation by a spin energy level of an electron split by a magnetic field. The EPR signal is recorded if the condition resulting from the difference in separation energy of spin energy levels E = hʋ = gµ_B_B in a magnetic field with induction B is met, where µB is a Bohr magneton, g is a proportionality parameter also called spectroscopic splitting factor, h is Planck’s constant and ʋ is the frequency of the electromagnetic wave resonantly fitted to the difference in energy levels [32]. The value of the g factor for the free electron, according to quantum electrodynamics calculations, amounting to 2.00232, and it changes due to the nature of the defect and its surroundings [33].

Resonance condition can be achieved by selecting different values of frequency ν and magnetic field B. Although due to equipment characteristics, measurements are usually taken based on linear changes of magnetic field B with the constant frequency of electromagnetic radiation ν = const. X band frequency is ~9.5 GHz. Microwave radiation absorption variation is registered and the first derivative of the signal is recorded. Obtained EPR signal is described by three parameters: resonance field B based on which g value is determined, intensity of EPR lines (I_EPR_) and line width ΔB. Double integral of EPR signal defines the concentration of paramagnetic centers in the sample and often is calculated in simplified way by using the equation I_int_ = I_EPR_ × ΔB^2^. This method allows to compare concentration of paramagnetic centers in different materials and even the number of spins in the sample (if spin standards are used). For investigated materials, number of spins was not determined due to the inability to measure the volume of the layer. Line width is related to the relaxation time and magnetic interaction in the sample. Value of g factor is closely connected with quantity of spin-orbit coupling and it is often anisotropic in the low symmetry systems. Additionally, g factor value depends on local environment symmetry, ligand types and distance and type of paramagnetic ions [34,35].

**Figure 7:**
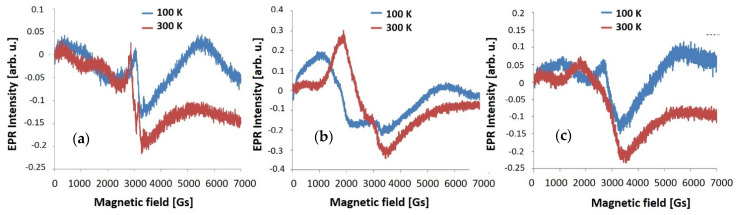
The EPR spectra measurement at 100 K and 300 K of (**a**) as-deposited; (**b**) heated at 300 °C and (**c**) heated at 450 °C, single Al_2_O_3_ layer produced by ALD method.

**Figure 8:**
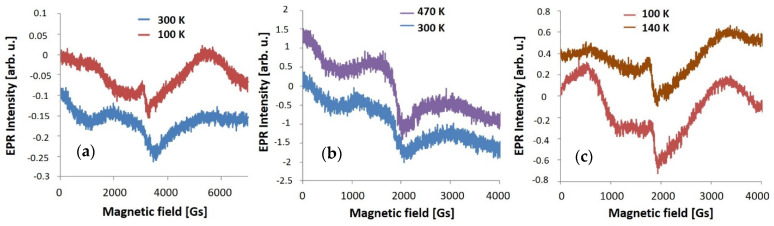
The EPR spectra measurement at different temperature of (**a**) as-deposited; (**b**) heated at 300 °C and (**c**) heated at 450 °C, single ZnO layer produced by ALD method.

EPR spectra are presented in Figure 7 for Al_2_O_3_ and in Figure 8 for ZnO. The as-deposited Al_2_O_3_ layer is characterized by a relatively intense EPR line with the g = 2.12 and a low-intensity line with g = 1.05, most likely related to oxygen defects. For T300 Al_2_O_3_, the line g = 1.97 and g = 2.23 are observed. The T450 Al_2_O_3_ exhibits the same g = 1.97 line with a very low intensity and a shifted line at g = 2.78. This reveals that the highest number of paramagnetic centers as defects occurs in Al_2_O_3_ layer after annealing at 300 °C.

All ZnO layers are characterized by EPR lines with a g value 2.13 and g = 2.02. In addition, a low-field line appeared with the different g values for each sample: g = 2.71 for as-deposited ZnO, g = 3.01 for T300 ZnO and g = 3.89 for T450 ZnO. The line with g = 1.88 appeared for all ZnO layers, although its intensity is small and comparable for all samples. Similarly, to Al_2_O_3_ layers, a low intense and wide g line about 1.06, which can be associated with oxygen defects, can be found. The EPR line with g = 1.06 is observed only in samples measured in ambient conditions or if the samples were kept for some time in atmosphere containing oxygen. Atmospheric oxygen is the cause of many defects on the sample surface, including point defects, such as vacancies. Therefore the type of defects was not identified, however, their total number V_Oatm_ was determined. The Al_2_O_3_/ZnO double layers deposited at 200 °C are characterized by very weak lines with g = 2.09, g = 3.89 and the line associated with oxygen defects g = 1.08. On the other hand, when sample is heated at 450 °C, two overlapping broad lines with g = 2.08 and 2.48, and two weak lines with g = 1.94 and 1.85 are visible. Observed EPR spectrum for various layers is in agreement with those described in the literature, especially for the observed oxygen vacancies for g value ~2 [36,37]. The g values for all layers are collected in Table 2.

The temperature dependence was not analyzed quantitatively due to the weak and overlapping EPR lines. The high-field line with g = 1.05 is related to defects caused by atmospheric oxygen. Samples kept in an inert atmosphere, such as pure nitrogen gas, should not exhibit this line. A strong absorption of microwaves for the double Al_2_O_3_/ZnO layer suggests a large number of defects in the form of hydrogen bonds. The g values in the range of 1.96 to 2.12 can be attributed to various ZnO defects caused by other ions or vacancies such as V_Zn_^−^ or V_O_^+^ which become visible in this range.

## 4. Conclusions

In this paper, the influence of conditioning temperature on defects in the Al_2_O_3_ and ZnO layers grown by atomic layer deposition was investigated. The bright field TEM and SAED analysis revealed that as-deposited ZnO exhibits hexagonal wurtzite ordering but the material is ordered in ultrafine grains. During post-treatment at 450 °C, the quality of the layer improves as grains coalesce into larger structures. On the other hand, the aluminum oxide layer is mostly amorphous. This does not change even after high temperature annealing. The STEM line scan analysis indicated that the bright line formed after annealing on the Al_2_O_3_/Si interface can be attributed to aluminum atoms diffusing into silicon. XPS analysis of as-deposited aluminum oxide indicated that it is non-stoichiometric in nature, the Al/O ratio is equal 0.78. Although, the ratio drops to 0.74 for Al_2_O_3_ after annealing at 450 °C, however the layer surface does not reach full stoichiometry. On the other hand, the binding energy corresponding to the Al 2p_3/2_ component (BE 119 eV) increases for heated layer which may indicate an improvement in their crystallinity and slow transition to the α-Al_2_O_3_ phase. XPS analysis of ZnO layer confirmed that with temperature increasing the amount of zinc on the surface falls down, which reduces the Zn/O atomic ratio. It was also found that the Zn-O binding energy increases with the temperature but the spectra peaks get broader, which suggest increasing number of defects at the studied surface. It is worth noting that for pristine zinc oxide with good crystallinity, BE of the Zn 2p_3/2_ component should be 1022 eV. Such a difference may indicate recrystallization of 2-dimensional ZnO into hexagonal structures at elevated temperature. Moreover, the O 1s spectrum also revealed the presence of V_O_ defects on the surface. The amount of oxygen vacancies increases with temperature.

As evidenced by EPR data, the greatest number of paramagnetic centers, especially defects, occurs in the Al_2_O_3_ layer after heating up at 300 °C. In ZnO, the g values between 1.96 and 2.12 can be attributed to various ZnO defects caused by other ions while low-intensity, spread out 1.06 line, can be associated with V_O_ defects. Strong microwaves absorption by a double Al_2_O_3_/ZnO layer can be an evidence of a large number of defects in the form of hydrogen bonds.

Furthermore, upon annealing the diffusion of Al atoms from Al_2_O_3_ into silicon probably takes place. It should be highlighted that neither the morphology nor the stoichiometry of the amorphous aluminum oxide layer improved when heated to 450 °C. The annealing process also does not ameliorate the layer’s defect, as shown by EPR results. At this point, it can be stated that post-treatment heating after Al_2_O_3_ and ZnO deposition by ALD method is not beneficial, particularly for the application in heterojunction p-type Cz-Si/Al_2_O_3_/n-type ZnO/AZO solar cells.

Although heating to temperatures as high as 300 and 450 °C is not used in practice with Al_2_O_3_/ZnO layers deposited by the ALD method, the present work shows how this temperature range affects the material parameters of the structure which allows for precise planning of the high-temperature processes and its influences on the above presented layers.

## Figures and Tables

**Table 1:** The resistivity of the ZnO layers deposited by ALD method directly on glass.

	As Deposited 200 °C	Annealed 300 °C	Annealed 450 °C
	Resistivity [Ωcm]		
ZnO	6.8 × 10^−3^	7.2 × 10^−3^	23.2 × 10^−3^

**Table 2:** The value of the g factor of Al_2_O_3_ and ZnO layers.

Layer/Process	As-Deposited	Annealed at 300 °C	Annealed at 450 °C
	g (-)	g (-)	g (-)
Al_2_O_3_	1.05	1.97	1.97
	2.12	2.23	2.78
ZnO	1.88	1.88	1.88
	2.02	2.02	2.02
	2.13	2.13	2.13
	2.71	3.01	3.89

## Data Availability

Not applicable.
