# Correction: Gawlińska-Nęcek et al. Influence of Conditioning Temperature on Defects in the Double Al_2_O_3_/ZnO Layer Deposited by the ALD Method. *Materials* 2021, *14*, 1038

**DOI:** 10.3390/ma19071384

**Published:** 2026-03-31

**Authors:** Katarzyna Gawlińska-Nęcek, Mateusz Wlazło, Robert Socha, Ireneusz Stefaniuk, Łukasz Major, Piotr Panek

**Affiliations:** 1 Institute of Metallurgy and Materials Science PAS, Reymonta 25, 30-059 Krakow, Poland; l.major@imim.pl (Ł.M.); p.panek@imim.pl (P.P.); 2 CBRTP—Research and Development Center of Technology for Industry, Ludwika Waryńskiego 3A, 00-645 Warszawa, Poland; mateusz.wlazlo@cbrtp.pl (M.W.); ncsocha@cyf-kr.edu.pl (R.S.); 3 Institute of Catalysis and Surface Chemistry PAS, Niezapominajek 8, 30-239 Krakow, Poland; 4 Center of Teaching Technical and Natural Sciences, University of Rzeszow, Pigonia 1, 35-959 Rzeszow, Poland; istef@ur.edu.pl

In the original publication [1], there were mistakes in Figures 5 and 6, as well as in the legend for Figure 5. The corrected Figure 5 and Figure 6, together with the corrected legend for Figure 5, are presented below. The authors state that the scientific conclusions are unaffected. This correction was approved by the Academic Editor. The original publication has also been updated.

## Figures and Tables

**Figure 5:**
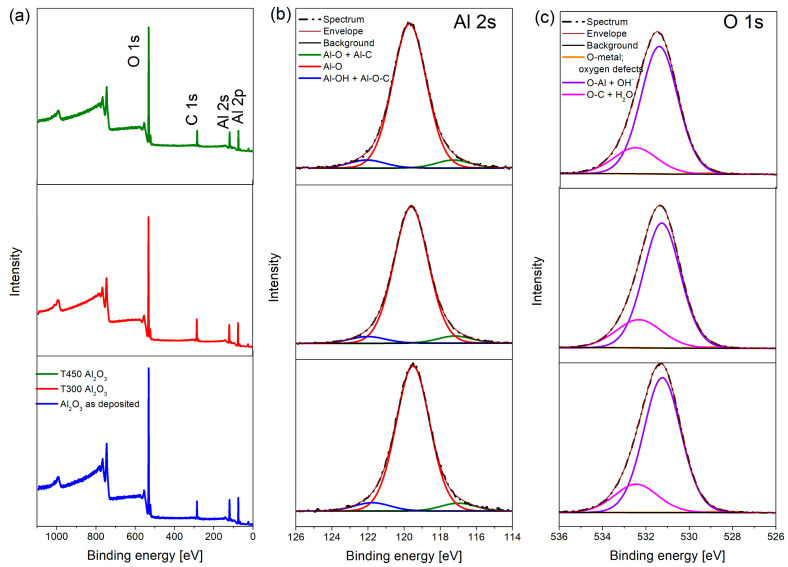
Al_2_O_3_ XPS: (**a**) survey spectra; (**b**) Al 2s spectra; (**c**) O 1s spectra, collected as deposited (**bottom**), after heating at 300 °C (**middle**), and after heating at 450 °C (**top**).

**Figure 6:**
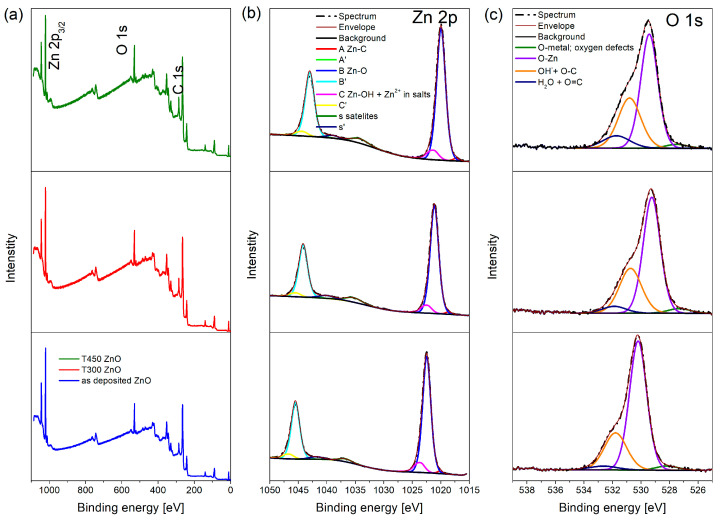
ZnO XPS: (**a**) survey spectra; (**b**) Zn 2p spectra; (**c**) O 1s spectra, collected as deposited (**bottom**), after heating at 300 °C (**middle**), and after heating at 450 °C (**top**).
